# Increased prevalence of depression and anxiety among adults initiating antiretroviral therapy during the COVID-19 pandemic in Shinyanga region, Tanzania

**DOI:** 10.1186/s12981-023-00534-y

**Published:** 2023-06-10

**Authors:** Solis Winters, Amon Sabasaba, Carolyn A. Fahey, Laura Packel, Emmanuel Katabaro, Yudas Ndungile, Prosper F. Njau, Sandra I. McCoy

**Affiliations:** 1 School of Public Health, University of California, Berkeley, 2121 Berkeley Way, Berkeley, 94704 USA; 2 Health for a Prosperous Nation, Dar es Salaam, Tanzania; 3 School of Public Health, University of Washington, Seattle, USA; 4 Ministry of Health, Dodoma, Tanzania

**Keywords:** HIV, Mental health, Depression, Anxiety, COVID-19, Syndemic

## Abstract

**Background:**

Concerns about the interconnected relationship between HIV and mental health were heightened during the COVID-19 pandemic. This study assessed whether there were temporal changes in the mental health status of people living with HIV presenting for care in Shinyanga region, Tanzania. Specifically, we compared the prevalence of depression and anxiety before and during COVID-19, with the goal of describing the changing needs, if any, to person-centered HIV services.

**Methods:**

We analyzed baseline data from two randomized controlled trials of adults initiating ART in Shinyanga region, Tanzania between April-December 2018 (pre-COVID-19 period, n = 530) and May 2021-March 2022 (COVID-19 period, n = 542), respectively. We compared three mental health indicators that were similarly measured in both surveys: loss of interest in things, hopelessness about the future, and uncontrolled worrying. We also examined depression and anxiety which were measured using the Hopkins Symptom Checklist-25 in the pre-COVID-19 period and the Patient Health Questionnaire-4 in the COVID-19 period, respectively, and classified as binary indicators per each scale’s threshold. We estimated prevalence differences (PD) in adverse mental health status before and during the COVID-19 pandemic, using stabilized inverse probability of treatment weighting to adjust for underlying differences in the two study populations.

**Results:**

We found significant temporal increases in the prevalence of feeling ‘a lot’ and ‘extreme’ loss of interest in things [‘a lot’ PD: 38, CI 34,41; ‘extreme’ PD: 9, CI 8,12)], hopelessness about the future [‘ a lot’ PD: 46, CI 43,49; ‘extreme’ PD: 4, CI 3,6], and uncontrolled worrying [‘ a lot’ PD: 34, CI 31,37; ‘extreme’ PD: 2, CI 0,4] during the COVID-19 pandemic. We also found substantially higher prevalence of depression [PD: 38, CI 34,42] and anxiety [PD: 41, CI 37,45].

**Conclusions:**

After applying a quasi-experimental weighting approach, the prevalence of depression and anxiety symptoms among those starting ART during COVID-19 was much higher than before the pandemic. Although depression and anxiety were measured using different, validated scales, the concurrent increases in similarly measured mental health indicators lends confidence to these findings and warrants further research to assess the possible influence of COVID-19 on mental health among adults living with HIV.

*Trial Registration* NCT03351556, registered November 24, 2017; NCT04201353, registered December 17, 2019.

## Background

Central to the achievement of UNAIDS’ Fast Track strategy to end the HIV/AIDS epidemic is support for people living with HIV (PLHIV) to engage in lifelong HIV care and antiretroviral therapy (ART) [1, 2]. Nevertheless, decades of research have established the deleterious effects of mental health conditions such as depression and anxiety on HIV care. In particular, PLHIV with depression are less likely to initiate HIV care [3], remain engaged in care [4, 5], and achieve viral suppression [4, 6, 7]. A systematic review of studies in sub-Saharan Africa found that HIV-positive individuals with significant depression symptoms had a 55% lower odds of achieving ART adherence compared to those without significant depression symptoms [3]. In addition, HIV and many mental health disorders share overlapping risk factors such that some conditions, including depression [8–10] and suicidal ideation [11], may be more prevalent among PLHIV than in the general population [12]. In East Africa, for example, the prevalence of depression among PLHIV is nearly 40% [13]; and even four decades into the HIV epidemic, HIV stigma remains rampant with significant negative impacts on mental health and HIV outcomes [14]. For these reasons, calls for the integration of HIV and mental health services in HIV primary care have become increasingly urgent [15, 16].

Concerns about HIV care initiation and retention among PLHIV were heightened at the advent of the COVID-19 pandemic, which had the potential to undermine global successes in HIV epidemic control via disruption of HIV care and access to ART, avoidance of HIV care due to fear of exposure to COVID-19, supply chain disruptions, and the possibility of increasing HIV incidence via secondary transmissions from those with detectable HIV viremia [17–23]. The COVID-19 pandemic may have been particularly detrimental to programs providing HIV testing and linkage to care [21, 24, 25]. For example, in South Africa, COVID-19 lockdowns were associated with an estimated 47.6% and 46.2% decrease in HIV testing and ART initiations, respectively [21].

The COVID-19 pandemic may also have negatively influenced HIV epidemic control through worsened mental health conditions. Within the first few months of the pandemic, the harmful consequences of COVID-19 and its associated control measures (.eg.., lockdowns, social distancing) on mental health in the general population were recognized; these trends were supported in subsequent population-level studies [26]. These harms were thought likely to be exacerbated among PLHIV, a population with unique psychosocial vulnerabilities related to their HIV status; however, the pandemic’s effects on the mental health of PLHIV is relatively understudied among those initiating HIV care in sub-Saharan Africa.

Thus, to understand whether there were temporal changes in the mental health status of PLHIV presenting for care in Tanzania, we leveraged baseline data from two linked trials evaluating an economic incentive intervention for increasing retention in care [27–29]. Our hypothesis-generating analysis compared the prevalence of depression and anxiety among ART initiates before and during COVID-19, with the goal of describing the changing needs, if any, to person-centered HIV services including mental health and psychosocial support.

## Methods

### Data

We analyzed baseline survey data from two randomized controlled trials (RCT) of adults initiating ART in the same region of Tanzania before and during COVID-19, respectively. The first RCT was conducted at four HIV primary care facilities in Shinyanga region, Tanzania. Adults 18 years or older, living with HIV, who had started ART no more than 30 days before enrolment were eligible for inclusion. Patients known to be temporarily in-transit and those facing a language barrier were excluded [27]. Baseline data were collected from 530 adults living with HIV between April-December 2018, prior to the start of the COVID-19 pandemic, including demographic, health, and health care information.

The second RCT was implemented at 32 HIV primary care clinics across four regions in Lake Zone, Tanzania—Shinyanga, Geita, Kagera, and Mwanza. Individuals 18 years or older living with HIV, who initiated ART at least 30 days before enrolment in the study, had access to a mobile phone, and who had no plans to transfer to a different HIV facility in the 12 months following enrolment were eligible for inclusion into the study [28]. As in the first RCT, patients facing a language barrier were also excluded. Baseline data were collected from 542 adults living with HIV between May 2021 and March 2022, over a year into the COVID-19 pandemic. This analysis was restricted to participants enrolled at eight clinics in Shinyanga region; participants in Geita, Kagera, and Mwanza were excluded to increase comparability between the two cohorts.

### Mental health indicators

In the pre-COVID-19 period, depression and anxiety symptoms were measured using the Hopkins Symptom Checklist-25 (HSCL-25) [30]. The first part of the scale includes 10 items related to anxiety symptoms and the second part includes 15 items related to depressive symptoms. Each item has four possible response options: ‘Not at all’, ‘A little’, ‘Quite a bit’, and ‘Extremely’, which are scored from 1 to 4, respectively. Average scores for the anxiety and depression symptoms subscales were calculated separately. Binary indicators for anxiety and depression were defined as an average score above 1.75 (out of a maximum of 4), which is the scale’s recommended threshold to signify a clinically relevant level of anxiety or depression symptoms. The HSCL-25 has been previously translated, validated, and used in East Africa: a 2002 study among pregnant women in Dar es Salaam recommended using a lower cutoff [31]; however, more recent studies among adult men and women living with HIV in East Africa have used and confirmed the reliability and validity of the conventional 1.75 cutoff [32–35].

In the COVID-19 period, depression and anxiety symptoms were measured using the Patient Health Questionnaire-4 (PHQ-4), a four-item scale comprised of the Patient Health Questionnaire-2 (PHQ-2) for depression and the Generalized Anxiety Disorder-2 (GAD-2) scale [36–38]. The PHQ-2 includes the first two items of the full nine-item scale, designed as a rapid approach to screening for depression. The scale asks about the frequency of depressed mood over the past two weeks. The GAD-2 is a similar two-item scale designed to screen for generalized anxiety disorder. It inquires about the frequency of anxious mood over the last 2 weeks. The four response options for all PHQ-4 questions are: ‘Not at all’, ‘Several days’, ‘More than half the days’, and ‘Nearly every day’, which are scored from 0 to 3, respectively. The scores for the two items in each subscale are added to obtain a total score between 0 and 6. Binary indicators for depression and anxiety were classified based on the optimal cutpoint of 3 points used to identify patients for further evaluation for depressive disorder or generalized anxiety disorder, respectively. The PHQ-4 scale has been previously translated and validated for use in the same region of Tanzania as our study [39].

We directly examined three mental health indicators that were similarly measured in both studies: loss of interest in things, hopelessness about the future, and uncontrolled worrying. Specifically, we compared the prevalence of people reporting having experienced these three problems ‘a lot’ or an ‘extreme amount’ in the last 2 weeks, where ‘a lot’ includes anyone who responded ‘Quite a bit’ or ‘More than half the days’ (second highest frequency responses on HSCL-25 and PHQ-4, respectively) and an ‘extreme amount’ includes anyone who responded ‘Extremely’ or ‘Nearly every day’ (highest frequency responses on HSCL-25 and PHQ-4, respectively) in the periods before and during COVID-19, respectively. We also compared the prevalence of depression and anxiety overall, as classified per each scale’s optimal cutpoint.

### Statistical analysis

Although the study design prevents causal interpretation of our findings, our analyses were motivated by causal questions–in particular, the possible influence of the COVID-19 pandemic on mental health and HIV care. Participants in both cohorts were enrolled at HIV primary care clinics in the same region and recruited using the same study inclusion criteria; however, it is possible that there are underlying differences in the two study populations that could influence the findings. Thus, to adjust for unmeasured confounders we employed a quasi-experimental strategy. Specifically, we estimated prevalence differences in mental health before and during the COVID-19 pandemic using stabilized inverse probability of treatment weighting (IPTW). IPTW creates a pseudo population by weighting individuals such that observed confounders are equally distributed across exposed (during COVID-19) and unexposed (pre-COVID-19) groups. Weights were defined as 1/propensity score for the exposed group and 1/(1—propensity score) for the unexposed group. Propensity scores were estimated using a logistic regression model with known predictors of mental health selected a priori including age, sex, primary language, education, marital status, household head, overall health, and work status as covariates [40, 41].

### Study characteristics

Participants in the pre- and COVID-19 periods were relatively similar in terms of age, sex, marital status, level of education, and employment status (Table 1). Study participants were in their mid-thirties on average and slightly over half were female. About 60% of participants were married or in a steady partnership. Less than 15% of participants completed schooling beyond primary school. Over half of participants were employed. As expected, self-reported overall health of the participants surveyed during COVID-19 was worse on average than those surveyed before the beginning of the pandemic. There was an approximately 15 percentage-point difference in the both the proportion of participants who reported Swahili as their primary language and the proportion who identified as head of household.

**Table 1:** Socio-demographic characteristics of ART initiates before and during the COVID-19 pandemic, Shinyanga region, Tanzania, 2018 & 2021–22

Characteristics	Pre-COVID-19 (2018) n = 530	COVID-19 (2021–2) n = 549
Age in years, mean (SD)	36 (10)	37 (12)
Female	330 (62%)	324 (59%)
Swahili as primary language^*^	244 (46%)	164 (30%)
Married or steady partner	288 (54%)	329 (60%)
Head of household	401 (76%)	319 (58%)
Highest education level		
No school	115 (22%)	156 (29%)
Some primary school	84 (16%)	92 (17%)
Completed primary school	255 (48%)	244 (45%)
More than primary school	76 (14%)	53 (10%)
Employed	309 (58%)	354 (65%)
Severe food insecurity	20 (4%)	5 (1%)
Overall health		
Excellent	59 (11%)	44 (8%)
* Very good*	173 (33%)	127 (23%)
Good	189 (36%)	156 (29%)
Fair	90 (17%)	161 (29%)
Poor	19 (4%)	59 (11%)

^*^Other language options included Sukuma, Haya, and Other

## Results

**Fig. 1:**
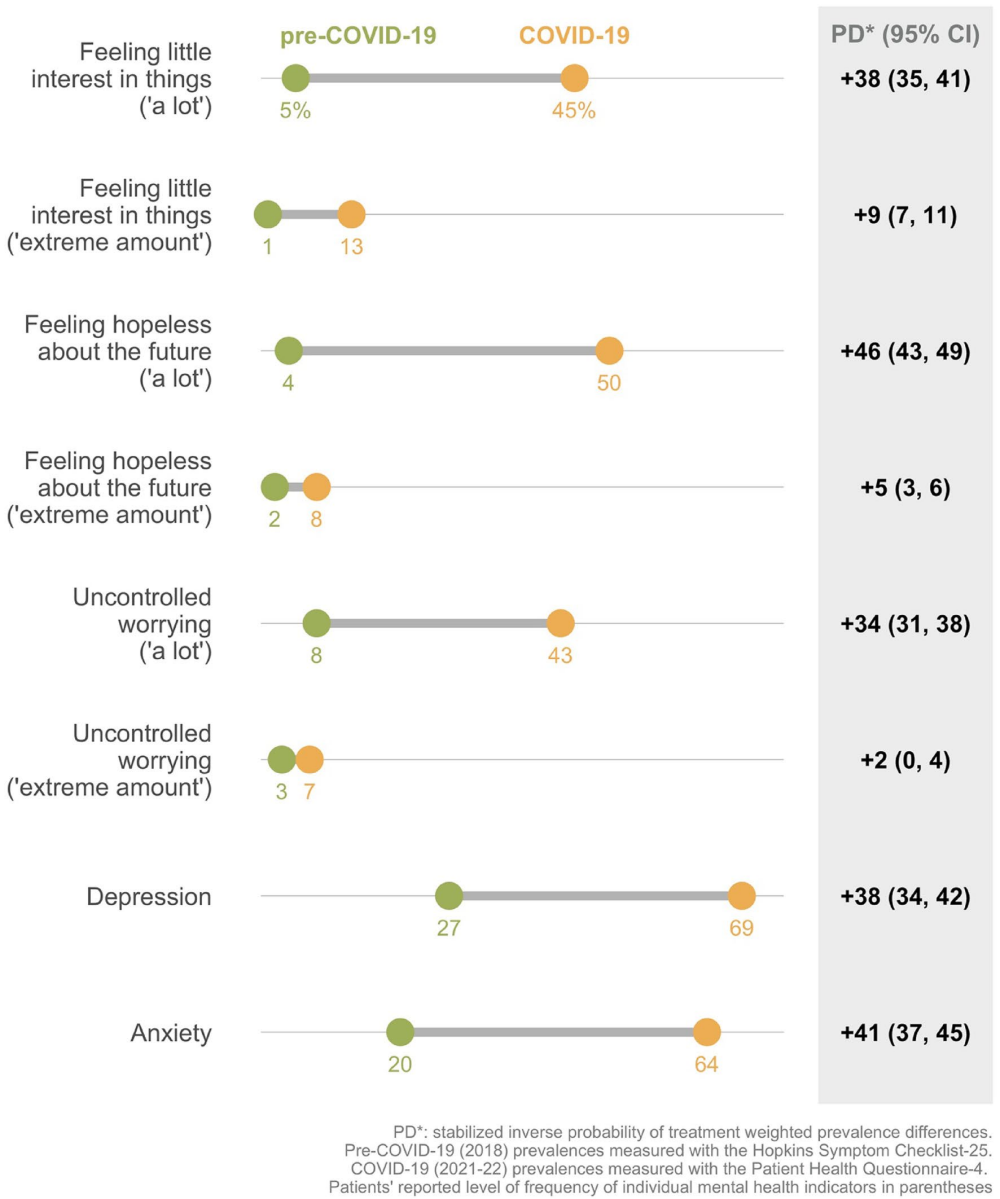
Weighted prevalence differences in mental health among ART initiates before and during the COVID-19 pandemic, Shinyanga region, Tanzania, 2018 & 2021–22

We compared the difference in prevalence of three depressive and anxious symptoms that were similarly asked in both studies; specifically, we estimated changes in participants reporting feeling ‘a lot’ or ‘extreme’ loss of interest in things, hopelessness about the future, and uncontrolled worrying (Fig. 1). We found higher prevalences for all three indicators during the pandemic compared to before. The weighted prevalence differences in those reporting feeling these moods ‘a lot’ ranged from 34 to 46 percentage points, respectively. Temporal differences in those feeling these moods an ‘extreme amount’ were attenuated, but statistically significant.

Overall, 27% of participants surveyed before the COVID-19 pandemic had symptoms of depression and 20% had symptoms of anxiety, per the HSCL-25 (Fig. 1). The prevalence of these mental health conditions among participants surveyed during the COVID-19 pandemic was substantially higher, with 69% participants considered to have symptoms of depression and 63% having symptoms of generalized anxiety disorder, per the PHQ-4. The weighted prevalence differences in mental health conditions comparing participants before and during the COVID-19 pandemic was 38 percentage points (95% CI 34, 42) for depression and 41 percentage points (95% CI 37, 45) for anxiety.

**Fig. 2:**
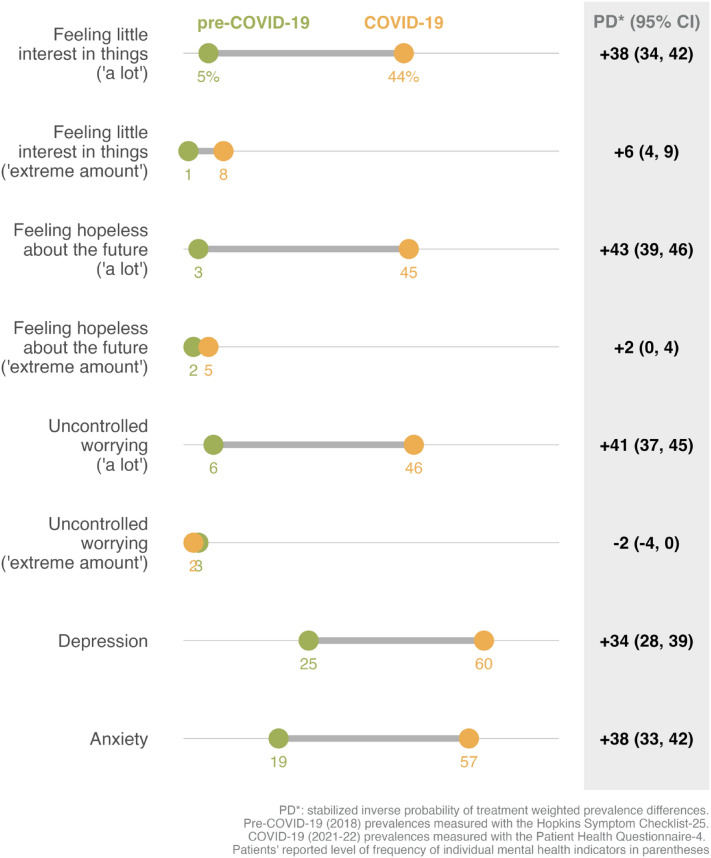
Weighted prevalence differences in mental health among ART initiates at Kahama Hospital, Kagongwa Dispensary, and Kambarage Health Center before and during the COVID-19 pandemic, Shinyanga region, Tanzania, 2018 & 2021–22

To ensure results were robust to differences in sample composition, we replicated the analyses using a subset of the sample limited to individuals recruited at the three clinics that were the same in both trials–Kahama Hospital, Kagongwa Dispensary, and Kambarage Health Center. The results were consistent with our main results, as seen in the Appendix (Table 2, Fig. 2).

## Discussion

Our study compared the prevalence of depression and anxiety among people initiating ART in Tanzania before and during COVID-19, with the goal to better understand and respond to the changing needs of PLHIV in the wake of the COVID-19 pandemic. After adjusting for underlying population differences in selected confounders through inverse probability of treatment weighting, a rigorous quasi-experimental approach, we found that the prevalence of depression and anxiety symptoms among those starting ART in Shinyanga during the pandemic was much higher than in 2018. More specifically, we found significant temporal increases in the prevalence of feeling ‘a lot’ or ‘extreme’ loss of interest in things, hopelessness about the future, and uncontrolled worrying. We also noted substantial increases in the prevalence of depression and anxiety of 38 and 41 percentage points, respectively. Although this analysis cannot definitively rule out the role of chance or bias, including unmeasured confounding, in these findings, our study is an early signal of possible heightened mental health issues among PLHIV beginning lifelong HIV care in Tanzania.

Our findings are consistent with other studies that show increased mental health illness among PLHIV during COVID-19. Evidence from three systematic reviews looking at the social and behavioral impacts of the COVID-19 pandemic on PLHIV in primarily high-income countries indicate higher levels of stress, anxiety, depression, and loneliness [42–44]. Primary reasons included concerns about the severity and impact of acquiring the virus while living with HIV and associated comorbidities; social isolation, which, for many, brought about memories of when they were initially diagnosed with HIV; fragile economic situation due to reductions in hours or job loss; and increased risk of HIV status disclosure and stigma [42–44]. The mental health impacts of the pandemic on PLHIV in sub-Saharan Africa remains limited; however, a qualitative study in rural Uganda highlighted the “double stress” of living with HIV during the COVID-19 pandemic. Respondents reported concerns about accessing ART, distress over inadvertent HIV status disclosure, fear of the severity of the virus for immunocompromised individuals, and exacerbated levels of poverty and economic stress [45]. In Tanzania, much uncertainty about the impacts of COVID-19 and a lack of clarity from country leadership with respect to how Tanzanians should respond given responses to the pandemic in neighboring countries and around the world may have increased anxiety for some, particularly for those who needed to regularly visit health facilities for ongoing treatment of chronic conditions, such as PLHIV. These additional stressors brought about and/or compounded by the COVID-19 pandemic are possible explanations for the mental health increases in this study, and underscore the importance of addressing mental health needs for PLHIV.

This analysis had several notable strengths. The focus on ART initiates provides valuable information about potential COVID-related challenges during a crucial window that can shape an individual’s trajectory for engagement in lifelong HIV care. PLHIV are highly vulnerable at the start of HIV care, often experiencing clinical symptoms of disease, coping with the burden of integrating HIV into their identity and possible stigma, and often economically vulnerable if they have left the labor force [46, 47]. Importantly, we used data from two similar cohorts: participants were recruited by the same research team, in the same region of Tanzania, using nearly identical protocols and eligibility criteria but spaced approximately three years apart. We further bolstered the comparability of these cohorts using a quasi-experimental weighting design to account for differences in the cohorts in several covariates.

Despite this rigorous approach, we cannot causally attribute the observed decline in mental health status to COVID-19 impacts. Differences in symptoms of depression and/or anxiety could also stem from unmeasured confounders, including potential temporal changes other than COVID-19 affecting ART initiates in Shinyanga region, such as political and social change. However, these results are hypothesis generating and provide justification for future surveillance and cohort studies of mental health among PLHIV. Another limitation of this research was the use of different scales to assess mental health status in each period, and potential sensitivity of our findings to the cutoffs used to classify depression and anxiety according to each scale. For this reason, we focused our analysis on the three individual items that were measured similarly across scales. The consistency of our results using the individual indicators lends credibility to the findings from the overall scales. Furthermore, the cutoff values for depression and anxiety have been used and validated in other East African contexts [32–35, 39]. These two scales have also been shown to be highly correlated, with a Pearson correlation coefficient of 0.88 [53].

A substantial worsening of mental health among PLHIV, regardless of the causal role of COVID-19, is concerning for several reasons. First, mental health conditions like depression and anxiety have significant physical, social, and economic consequences on individuals, families, and communities, independent of HIV. Furthermore, when experienced by PLHIV, these conditions are known to undermine an individual’s ability to initiate HIV care [3], remain on ART [4, 5], and achieve viral suppression [4, 6, 7]. When depression and anxiety are widespread among PLHIV in a population, they have the potential to unravel many of the successes of the global HIV response. The 2022 UNAIDS Global AIDS Update reports that the number of people on HIV treatment increased by only 1.47 million in 2021, compared to increases of greater than 2 million people in prior years–the smallest increase since 2009 [2]. This downward trajectory in the number of PLHIV receiving treatment coincides with economic strain due to the pandemic and reduced international assistance for the AIDS response from some major bilateral donors. While our study was conducted in a single region in Tanzania and the results cannot be widely extrapolated, our results in tandem with global epidemiologic and economic trends raise concerns about possible worsening mental health among PLHIV, and underscore the need for future research and policy initiatives to avoid further deceleration of progress to end the HIV epidemic.

Fortunately, there are a host of evidence-based interventions to support the mental health of PLHIV. Evidence from low- and middle-income countries (LMIC) broadly support the use of psychological interventions over pharmacological interventions [48, 49]. In particular, interventions incorporating cognitive behavioral therapy, positive coping skills, social support, venting, and problem-solving have proved most successful in improving mental health conditions and HIV outcomes [48–52]. The most effective interventions incorporate several of these components and are delivered in community- or group-based settings by lay or community health workers. To improve sustainability and acceptability and increase effectiveness, interventions should involve the target population in intervention design and directly respond to the needs of the individuals [48–50]. One example of a promising intervention to improve mental health and wellbeing of PLHIV is the Friendship Bench, a problem-solving therapy delivered on benches in primary care facilities by trained lay health workers, traditionally elderly women known as community “grandmothers” [52]. Another group-based intervention in Tanzania, “Sauti ya Vijana” (“The Voice of Youth”), which incorporated cognitive-based therapy, interpersonal psychotherapy, and motivational interviewing, showed improvements in mental health, ART adherence, and viral suppression among young PLHIV [51]. The results from our study, which show worsening mental health conditions among PLHIV during the COVID-19 pandemic, highlight the urgent need to integrate and scale-up mental health interventions such as these into HIV primary care.

## Conclusions

After applying a quasi-experimental weighting approach, the prevalence of depression and anxiety symptoms among PLHIV starting ART in Tanzania during COVID-19 was much higher than before the pandemic. Although overall measures of depression and anxiety were measured using different, validated scales, the concurrent increases in similarly measured mental health indicators lends confidence to these findings and warrants further research to assess the impact of COVID-19 on mental health among PLHIV. Contemporaneous with additional, rigorous research on the mental health status of PLHIV, program implementers may wish to consider whether enhanced mental health support via evidence-based interventions, such as those discussed above, are appropriate to adopt or bolster in the post-COVID pandemic period for the various populations of PLHIV they serve.

## Data Availability

The datasets used and/or analyzed during the current study are available from the corresponding author on reasonable request.

## See Table 2

**Table 2:** Socio-demographic characteristics of ART initiates at Kahama Hospital, Kagongwa Dispensary, and Kambarage Health Center before and during the COVID-19 pandemic, Shinyanga region, Tanzania, 2018 & 2021–22

Characteristics	Pre-COVID-19 (2018) n = 488	COVID-19 (2021–2) n = 211
Age in years, mean (SD)	36 (10)	36 (11)
Female	304 (62%)	122 (58%)
Swahili as primary language^*^	221 (45%)	102 (48%)
Married or steady partner	268 (55%)	124 (59%)
Head of household	370 (76%)	133 (63%)
Highest education level		
No school	113 (23%)	47 (22%)
Some primary school	78 (16%)	37 (18%)
Completed primary school	229 (47%)	94 (45%)
More than primary school	68 (14%)	32 (15%)
Employed	279 (57%)	144 (68%)
Severe food insecurity	20 (4%)	3 (1%)
Overall health		
Excellent	52 (11%)	21 (10%)
Very good	161 (33%)	58 (28%)
Good	180 (37%)	55 (26%)
Fair	79 (16%)	59 (28%)
Poor	16 (3%)	18 (9%)

* Other language options included Sukuma, Haya, and Other

## Abbreviations

AIDS: Acquired immunodeficiency syndrome
ART: Antiretroviral therapy
CI: 95% Confidence interval
COVID-19: Coronavirus disease 2019
GAD-2: Generalized anxiety disorder-2
HIV: Human immunodeficiency virus
IPTW: Stabilized inverse probability of treatment weighting
LMIC: Low- and middle-income countries
PHQ-2: Patient health questionnaire-2
PLHIV: People living with HIV/AIDS
RCT: Randomized controlled trial
UNAIDS: Joint United Nations Programme on HIV/AIDS
